# Improved Quantification, Propagation, Purification and Storage of the Obligate Intracellular Human Pathogen *Orientia tsutsugamushi*

**DOI:** 10.1371/journal.pntd.0004009

**Published:** 2015-08-28

**Authors:** Suparat Giengkam, Alex Blakes, Peemdej Utsahajit, Suwittra Chaemchuen, Sharanjeet Atwal, Stuart D. Blacksell, Daniel H. Paris, Nicholas P. J. Day, Jeanne Salje

**Affiliations:** 1 Mahidol-Oxford Tropical Medicine Research Unit, Faculty of Tropical Medicine, Mahidol University, Bangkok, Thailand; 2 Department of Medicine, University of Oxford, Oxford, United Kingdom; 3 Department of Biochemistry, University of Oxford, Oxford, United Kingdom; 4 Centre for Tropical Medicine and Global Health, Nuffield Department of Medicine, University of Oxford, Oxford, United Kingdom; University of Texas Medical Branch, UNITED STATES

## Abstract

**Background:**

Scrub typhus is a leading cause of serious febrile illness in rural Southeast Asia. The causative agent, *Orientia tsutsugamushi*, is an obligate intracellular bacterium that is transmitted to humans by the bite of a *Leptotrombidium* mite. Research into the basic mechanisms of cell biology and pathogenicity of *O*. *tsutsugamushi* has lagged behind that of other important human pathogens. One reason for this is that *O*. *tsutsugamushi* is an obligate intracellular bacterium that can only be cultured in mammalian cells and that requires specific methodologies for propagation and analysis. Here, we have performed a body of work designed to improve methods for quantification, propagation, purification and long-term storage of this important but neglected human pathogen. These results will be useful to other researchers working on *O*. *tsutsugamushi* and also other obligate intracellular pathogens such as those in the *Rickettsiales* and *Chlamydiales* families.

**Methodology:**

A clinical isolate of *O*. *tsutsugamushi* was grown in cultured mouse embryonic fibroblast (L929) cells. Bacterial growth was measured using an *O*. *tsutsugamushi*-specific qPCR assay. Conditions leading to improvements in viability and growth were monitored in terms of the effect on bacterial cell number after growth in cultured mammalian cells.

**Key results:**

- Development of a standardised growth assay to quantify bacterial replication and viability *in vitro*.
- Quantitative comparison of different DNA extraction methods.
- Quantification of the effect on growth of FBS concentration, daunorubicin supplementation, media composition, host cell confluence at infection and frequency of media replacement.
- Optimisation of bacterial purification including a comparison of host cell lysis methods, purification temperature, bacterial yield calculations and bacterial pelleting at different centrifugation speeds.
- Quantification of bacterial viability loss after long term storage and freezing under a range of conditions including different freezing buffers and different rates of freezing.

**Conclusions:**

Here we present a standardised method for comparing the viability of *O*. *tsutsugamushi* after purification, treatment and propagation under various conditions. Taken together, we present a body of data to support improved techniques for propagation, purification and storage of this organism. This data will be useful both for improving clinical isolation rates as well as performing *in vitro* cell biology experiments.

## Author Summary

Scrub typhus is a serious, neglected tropical disease that is endemic in large parts of Asia and northern Australia. It is caused by the bacterium *O*. *tsutsugamushi*, which is maintained in Leptotrombiculid mites, small arthropods that occasionally bite humans and transmit the disease. *O*. *tsutsugamushi* is an obligate intracellular bacterium, which means that it can only survive and grow when it is physically enclosed within a cell, both when it is living in its vector mite, and when it is living in the human or other mammalian host. This makes it difficult to work with in the laboratory, as it needs to be cultured together with host cells. This technical difficulty is one reason why our understanding of this human pathogen is less well-developed than for many other pathogens of equivalent incidence and severity. Here, we have performed a body of work that was designed to measure and improve methods for growing these bacteria in the laboratory, purifying the bacteria from their host cells without damaging them, and preserving bacteria for long periods of time by cryopreservation. This work will support future efforts to understand the basic science behind this and similar intracellular human pathogens.

## Introduction

Scrub typhus is a serious febrile illness of broad geographical diversity, endemic in the majority of rural Asia and northern areas of Australia. Clinical symptoms resemble that of a number of other tropical diseases including malaria, dengue, leptospirosis, and other bacterial infections, and rapid, unambiguous diagnosis is often unavailable [1]. Consequently it is difficult to know the exact distribution and prevalence of scrub typhus, but best estimates suggest that one billion people per year are at risk and one million people per year are infected. Recent epidemiological studies showed that scrub typhus is a leading cause of serious, under-reported non-malarial fever in rural Thailand, Laos, China and Myanmar [2–6]. It has recently been shown to be a leading cause of CNS infections in a hospital in Laos [7], and is associated with high miscarriage and poor neonatal outcome rates in pregnancy [8].

Scrub typhus is caused by the obligate intracellular Gram-negative bacterium, *Orientia tsutsugamushi*. This is a member of the *Rickettsiaceae* family, but differs from bacteria of the genus *Rickettsia* in important aspects of genome structure, morphology and phenotypic properties [9,10]. *O*. *tsutsugamushi* has been shown to infect a wide range of cell types *in vitro*, but *in vivo* studies report that it is largely localised to endothelial cells, monocytes and dendritic cells in infected humans [11–17].

One reason why research into the fundamental mechanisms of cellular infection by *Orientia tsutsugamushi* is less well characterised than those of other equivalent human pathogens is because of the technical difficulties and uncertainties associated with culturing this bacterium *in vitro*. Clinical isolation of this organism from infected patients presents a major difficulty, where best practice results in isolation rates of only about 40% of PCR-confirmed patients [18], and where bacterial growth and isolation typically requires 4 weeks of growth. This is a serious concern because it means that bacterial culture cannot be used as a diagnostic tool for clinical purposes, and because it reduces the opportunity to generate bacterial isolates for research into strain diversity and potential antibiotic resistance.

In this study we set out to measure and improve methods for the quantification, propagation, purification and long-term storage of *Orientia tsutsugamushi*. We were inspired by a set of elegant experiments performed by the group of Barbara Hanson in the 1980s, which measured the growth of *O tsutsugamushi* (then called *Rickettsia tsutsugamushi)* under a range of conditions [19], and we aimed to update and expand on those observations. It is our hope our observations will benefit researchers engaged in both basic cell biology research and applied clinical diagnostic research.

## Materials and Methods

### Mammalian cell culture

L929, a mouse fibroblast line, was cultured in Dulbecco’s modified Eagle’s medium (DMEM; Gibco BRL). The media was supplemented with 10% fetal bovine serum (FBS, GIBCO BRL) without antibiotic, unless stated otherwise. Monolayers of L929 were cultured in T25 or T75 cell culture flasks at 37°C in a humidified atmosphere containing 5% CO_2_. When the cells reached 80–100% confluence, they were ready to be infected with *O*. *tsutsugamushi* or to be subcultured into a new flask by trypsinisation.

Subculture by trypsinisation was performed as follows. The culture medium was discarded and the cell layer washed one time with 1X PBS. To disaggregate the cells, 1 ml of 0.25% Trypsin/EDTA (10x Trypsin/EDTA 1:250, PAA, diluted with PBS) was added to the flask and incubated at 37°C. Flasks were tapped gently to facilitate detachment from the flask. Complete detachment was achieved at 3 mins, and was monitored by microscopy. Culture medium at 3x the volume of trypsin was added to neutralize the enzyme and to disperse the cells. The cell suspension was centrifuged at 1000 xg for 5 mins. The cell pellet was collected and resuspended into new medium, and the cell quantity was determined using a Trypan Blue dye exclusion assay. Cells were diluted into fresh growth medium at a ratio of 1:10, and transferred to a new culture flask or grown in appropriate vessels (12-well or 24-well cell culture plates) at 37°C with 5% CO_2_. After 1 day a confluent cell layer was formed.

### Culturing *O*. *tsutsugamushi*

*O*. *tsutsugamushi* strain UT-76 (a Karp-like strain from Thailand) was propagated in T25 cell culture flasks containing a confluent monolayer of L929 cells which were grown in DMEM supplemented with 10% FBS at 35°C with 5% CO_2_. After 7 days of infection the cells were sub-cultured onto fresh L-929 cell monolayers. The infected flasks were harvested by scraping using a sterile inoculating loop in 1ml spent medium and disrupted to release intracellular bacteria by lysing in a bullet blender (BBX24B, Bullet Blender Blue, Nextadvance, USA) used at power 8 for 1 min. The lysed bacteria were added to a T25 flask (100 μl/flask) containing an uninfected L-929 monolayer at 70–100% confluence. Infected flasks were passaged at a ratio of 1:5 (original culture flask: subculture flask). Bacteria were sub-cultured for a total of <20 passages, where passage 0 was the original clinical isolate.

Antibiotics were only used in the experiment testing the effect of antibiotic addition. Here antibiotics were used at the following concentrations: chloramphenicol 150 μg/ml, penicillin G 100 μg/ml, penicillin G + streptomycin (premix from Sigma-Aldrich) 125 μg/ml + 200 μg/ml.

Cell lines and bacteria were tested for the presence of mycoplasma and confirmed to be mycoplasma-free using the VenorGeM PCR detection kit (Minerva biolabs).

### Bacterial quantification by qPCR

The DNA copy number of *O*. *tsutsugamushi* or L929 cells was measured using qPCR targeting the 47 kDa gene or cfd gene respectively. The primer and the TaqMan probe for the 47 kDa gene were as follows: 47 kDa FW, (5’-TCCAGAATTAAATGAGAATTTAGGAC-3’); 47 kDa RV (5’-TTAGTAATTACATCTCCAGGAGCAA-3’); and 47 kDa probe (5’-FAM-TTCCACATTGTGCTGCAGATCCTTC-TAMRA-3’). The primer and TaqMan probe for the cfd gene were as follows: cfd FW (5’- ACTGAGATCGCTTTTGGGTC-3’); cfd RV (5’- GGAGGGTAGGTGTATTGTAAGG-3’) and cfd probe (5’- 5HEX-CTGGGTTGGAGGTGTCTGTGGT-BHQ2–3’). The qPCR mixture was composed of 1X Platinum supermix (Platinum Quantitative PCR SuperMix-UDG, Invitrogen, USA) or 1X qPCRBIO Probe Mix (qPCR Probe Mix LO-ROX, PCR Biosystems, UK), 0.1 μM forward and reverse primers, 0.2 μM probe, sterile distilled water and 1 μl of extracted DNA. Real time PCR was performed in a 100 TM Thermal Cycle Instrument (Biorad, CFX96 real time system) using the following conditions: initial denaturation at 95°C for 2 min, followed by 45 cycles of denaturation at 95°C for 15 sec and combined annealing and extension at 60°C for 30 sec with the acquisition of fluorescence. The DNA copy number was calculated by comparison with a standard curve.

### DNA extraction techniques

DNA extraction of cell pellets containing *O*. *tsutsugamushi* was performed using four different methods: Alkaline lysis, the commercial Qiagen DNeasy kit, boiling in water or boiling in DMEM.

The protocol for DNA extraction by alkaline lysis (hotshot) was as follows. The cell pellet was resuspended in 20–80 μl alkaline lysis buffer (25 mM NaOH, 0.2 mM EDTA) and boiled at 95°C for 15–60 min. The sample was cooled to 4°C and neutralization buffer (40 mM Tris-HCl, pH 7–8) was added at an equal volume (20–80 μl). Smaller volumes were used where lower bacterial numbers were expected.

DNA extraction using the Qiagen DNeasy kit was performed following the manufacturer’s instructions. DNA extraction by boiling in water or DMEM was performed by resuspending cell pellets in 40 μl water or DMEM and boiling at 95°C for 30 min.

Extracted DNA samples were stored at-20°C as required. Where DNA extraction methods were being directly compared, identical cell pellets were resuspended in an equal final volume.

### Host cell release from cell culture plates/flasks

Infected host cells grown in 12- or 24-well culture plates were harvested for DNA extraction using three different methods: trypsinisation, RIPA buffer and scraping.

For trypsinisation, cells were washed once with PBS and resuspended in 1 ml 0.25% Trypsin/EDTA (10x Trypsin/EDTA 1:250, PAA, diluted with PBS). Cell detachment was monitored by microscopy, and fully detached cells were transferred to 1.5 ml microcentrifuge tubes. Samples were centrifuged at 20,238 xg for 3 min, and the pellet processed for DNA extraction.

Cells released using RIPA buffer were treated as follows. First, the supernatant was removed and either discarded or collected by centrifugation. Then, 300 μl (24 well plate) or 500 μl (12 well plate) of RIPA buffer (Sigma) was added into each well and incubated at room temperature (RT) or 37°C for 3–5 min. Detached cells were monitored under the microscope, and fully detached samples were transferred to a 1.5 ml microcentrifuge tube and centrifuged at 20,238 xg for 3 mins. The pellet was collected for DNA extraction.

Samples prepared by scraping were processed as follows. A sterile plastic inoculating loop was used to remove attached infected cells from the bottom of a 12- or 24-well culture plate, and cells were resuspended directly in the original growth media. The entire volume of resuspended cells was transferred to a 1.5 ml microcentrifuge tube, spun at 20,238 xg for 3 min, and the supernatant removed. Wells were inspected after scraping to assess the efficiency of the process.

### Bacterial purification techniques

Bacteria were purified from host cells after 7 days growth. Host cells were released from the surface of T25 or T75 culture flasks by scraping with a sterile inoculating loop, then the total volume was transferred to a sterile 15 ml falcon tube and centrifuged at 4,000 rpm for 10 mins. The cell pellet was collected and resuspended in 1–2 ml spent medium or new medium. Host cells were lysed using either a vortex or a bullet blender (BBX24B, Bullet Blender Blue, Nextadvance, USA) either without beads or with 0.2 mm glass beads (Life Science AP, Thailand) or with 0.9–2.0 mm stainless steel beads (Next Advance, USA). Unless stated in the text, lysis was done using the bullet blender at power 8 for 1 min with no beads added. Lysis by bullet blender was performed using Eppendorf Safe Lock tubes (Eppendorf, USA) and lysis by vortex was done using regular 1.5 ml microcentrifuge tubes. Lysed cells were centrifuged once at 50 xg for 3 min to pellet residual host cells and large cell debris. The supernatant containing released bacteria was transferred to a clean 1.5 ml microcentrifuge tube, and this was centrifuged at 20,238 xg for 3 mins to pellet cell-free bacteria. Pellets were collected and resuspended in different buffers for further experiments. Purification was performed at room temperature unless stated otherwise.

### Bacterial storage methods

*O*. *tsutsugamushi* purified as described above were treated in a number of different ways in order to assess viability after short- and long-term storage. For short-term storage, purified bacteria were resuspended in either sucrose-phosphate-glutamate (SPG) buffer (0.218 M sucrose, 3.76 mM KH_2_PO_4_, 7.1 mM KH_2_PO_4_, 4.9 mM monosodium L-glutamic acid), PBS, PBS + 10% BSA, DMEM + 10% FBS or water, and stored at room temperature for 30 mins or 120 mins before being grown on L929 cells in 24 well plates and assessed for growth after 7 days.

For long-term storage purified bacteria or bacteria in intact host cells were pelleted and resuspended in a range of different buffers: SPG, SPG + 15% glycerol, SPG + 10 mM MgCl_2_, SPG + 10 mM MgCl_2_ + 15% glycerol, RPMI, RPMI + 15% glycerol, or 90% FBS + 10% glycerol. All samples were transferred to a 1.5 ml cryovial and placed directly in the-80°C freezer and stored for 2 hours, then thawed at 37°C for 5 min. Samples were infected onto fresh monolayers of L929 in 24-well plates and assessed for growth after 7 days.

### Bacterial freezing methods

Experiments were performed to compare the effect of freezing and thawing at different speeds. Purified bacteria were pelleted and resuspended in SPG buffer. Samples were transferred to a cryovial and frozen and thawed under different conditions. For freezing, cells were either frozen fast (placed in a dry-ice gel for 10 min before being transferred to-80°C freezer) or slow (by placing samples in an CoolCell LX (Biocision) alcohol-free freezing chamber directly in the-80°C freezer, which freezes samples at a rate of ~ 1°C per minute). For thawing, cells were either thawed fast (placed in a 37°C water bath until thawed) or slow (placed one ice until thawed completely). Bacteria were then seeded onto L929 cells and assessed for growth after 7 days.

### Immunofluorescences and confocal microscopy

Infected L929 cells, which had been lysed in different ways, were assessed by fluorescence confocal microscopy in order to determine to effect of lysis on morphology. Two μl of lysed samples were fixed on the 12-well slide with 100% acetone for 10 mins at-20°C then left to dry completely. Slides were then incubated in a humidified slide chamber at room temperature with 1/100 anti-*O*. *tsutsugamushi* primary monoclonal antibody (made in house) for 30 mins, washed three times with PBS and incubated with 1/400 Alexafluor 488 anti-mouse secondary antibody (Invitrogen, USA) for 30 mins. Evans blue was included with the secondary antibody at a dilution of 1/200. The samples were washed three times with PBS, then mounted using Vectamount (Vecter Laboratories, Burlinggame, CA, USA) which contained the blue nuclear intercalating counterstain DAPI (4′,6-diaminidimo-2-phenylindole). Samples were examined under confocal laser scanning microscope (Zeiss LSM 700).

### Accession numbers for genes and proteins

47 kDa antigen gene from *O*. *tsutsugamushi* (strain Ikeda): OTT_1319/ID 6337858 cfd gene from *Mus musculus*: ID 11537

## Results and Discussion

### Developing an assay to quantify bacterial viability and growth

First, we aimed to develop a standardised assay that would generate accurate and reliable quantitative information about the number of viable cells in a bacterial sample. This assay would be used to analyse two types of experiments. (1) We would treat purified bacterial samples in different ways and compare the effects of these treatments on bacterial viability and (2) we would quantify the growth of bacteria under different growth conditions. When deciding how to assess viability and growth for these experiments we rejected fluorescence microscopy-based assays, such as the syto9/propidium iodide live/dead reagents, because we wanted to specifically measure the ability of viable *O*. *tsutsugamushi* to invade and replicate within host cells, and we could not exclude the possibility that microscopy-based assays would score some bacteria as live, that were structurally intact but metabolically dormant or inactive. Therefore, we decided to develop an assay based on active uptake and replication of bacterial cells.

We opted for qPCR to measure bacterial copy numbers. As an obligate intracellular bacterium it is not possible to directly assess the bacterial copy number using light absorption (OD) measurements as would be done for other model organisms such as *E*. *coli*. Plaque-based assays could have been used [20–22] but these are difficult to scale up to test large numbers of conditions and biological repeats. Finally, it would have been possible to quantify bacterial levels using light microscopy techniques such as Giemsa staining or acridine orange as has been done previously [23], but the accuracy and reproducibility of this method is low. We therefore quantified *O*. *tsutsugamushi* levels by extracting total DNA and measuring the exact genome copy number using a primer/probe set specific to a short region within the *O*. *tsutsugamushi* 47kDa *Htra* single copy gene. A similar approach has been used to track the growth of spotted fever group *Rickettsiae* in cultured cells [24].

It would be desirable to measure a complete growth curve over 1–2 weeks for each different condition, as this would give complete information about the degree of uptake and dynamics of intracellular replication of the bacteria. This approach, however, would require a prohibitive number of samples since each condition would need to be measured multiple times to account for biological variation. We therefore examined whether a single time point could give an accurate indication of the number of viable bacteria and the degree of intracellular growth. In order for this to be a robust measurement of uptake and growth the bacterial copy number at a single time point must be linearly proportional to the number of bacteria added at the time of infection.

**Fig 1:**
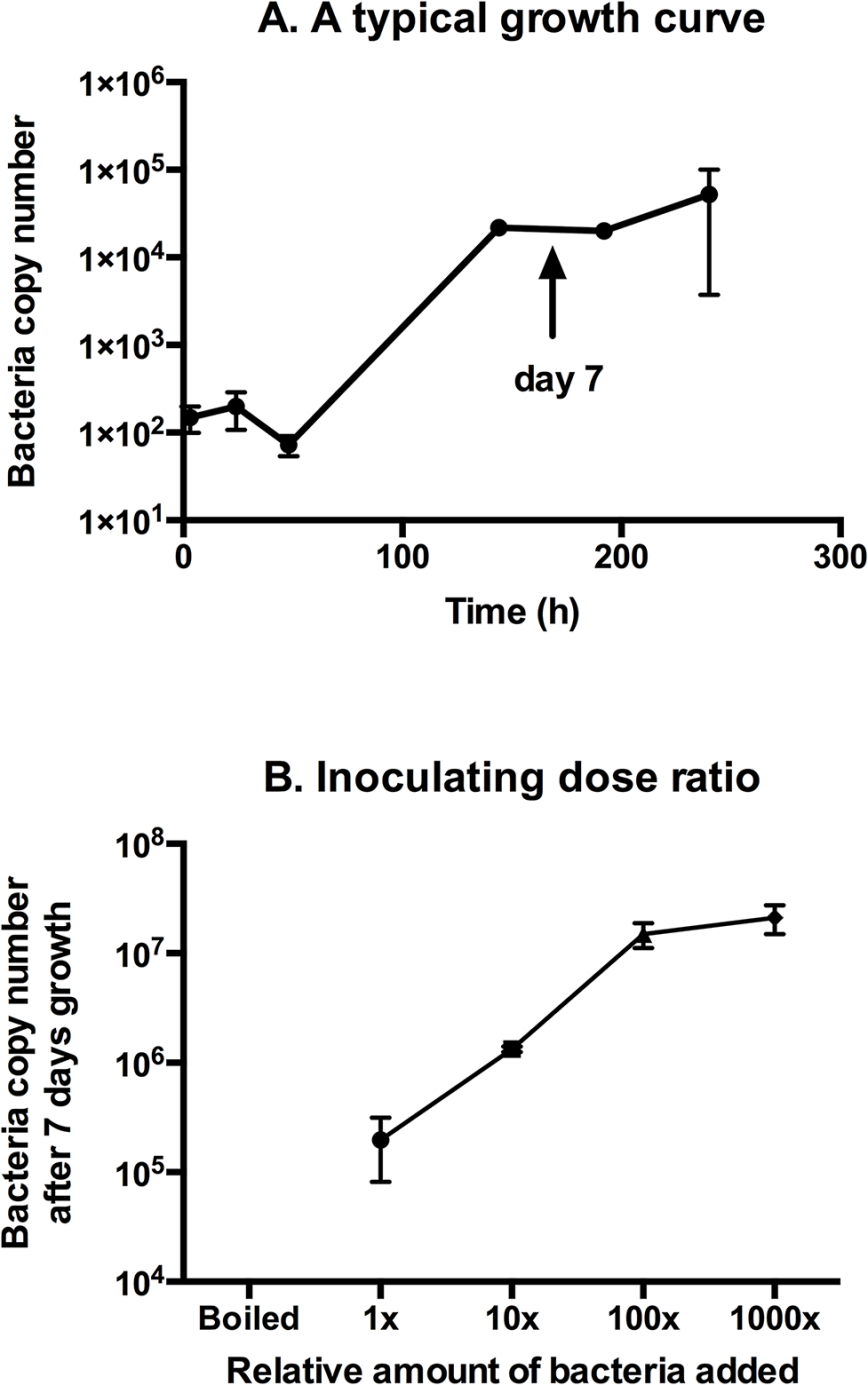
Panel A. A typical growth curve. An arrow indicates day 7, the time point used in subsequent experiments. Panel B. Inoculating dose ratio. This figure shows the bacterial count after 7 days, from different relative amounts of bacteria added at day 0. A boiled sample was included, which was added at 1x relative amount.

Fig 1A shows a typical growth curve of *O*. *tsutsugamushi* in cultured mouse fibroblast (L929) cells. After 7 days, the bacteria have achieved a substantial level of replication, and this was the time point we selected for all subsequent experiments. In order to validate this choice, we measured the growth after 7 days for a single batch of *O*. *tsutsugamushi* inoculated onto host cells at different concentrations. Fig 1B shows that the bacterial copy number after 7 days is roughly proportional to the relative number of bacteria added at the time of infection within a certain range (Here, 1x10^5^–1x10^7^ copy numbers). This is probably because when the bacteria level gets too high they saturate the L929 host cells available for infection and therefore no further bacterial growth is permitted. Fig 1B also demonstrated that bacteria boiled for 30 mins produced no detectable bacteria after 7 days of growth.

In summary, this shows that a single measurement after 7 days of isolate growth can be used to determine the relative number of bacteria available for uptake and growth in L929 host cells and thus to accurately compare conditions for bacterial treatment and growth. Infected cells were always washed 3 hours after infection in order to remove non-infectious extracellular bacteria. In order to account for differences in the exact number of bacteria being used in different conditions within an experiment, a portion of the inoculating bacteria was always retained for each experimental condition. This was quantified by qPCR and used to normalise the 7-day growth between conditions.

### Optimising quantification of *O*. *tsutsugamushi*

**Fig 2:**
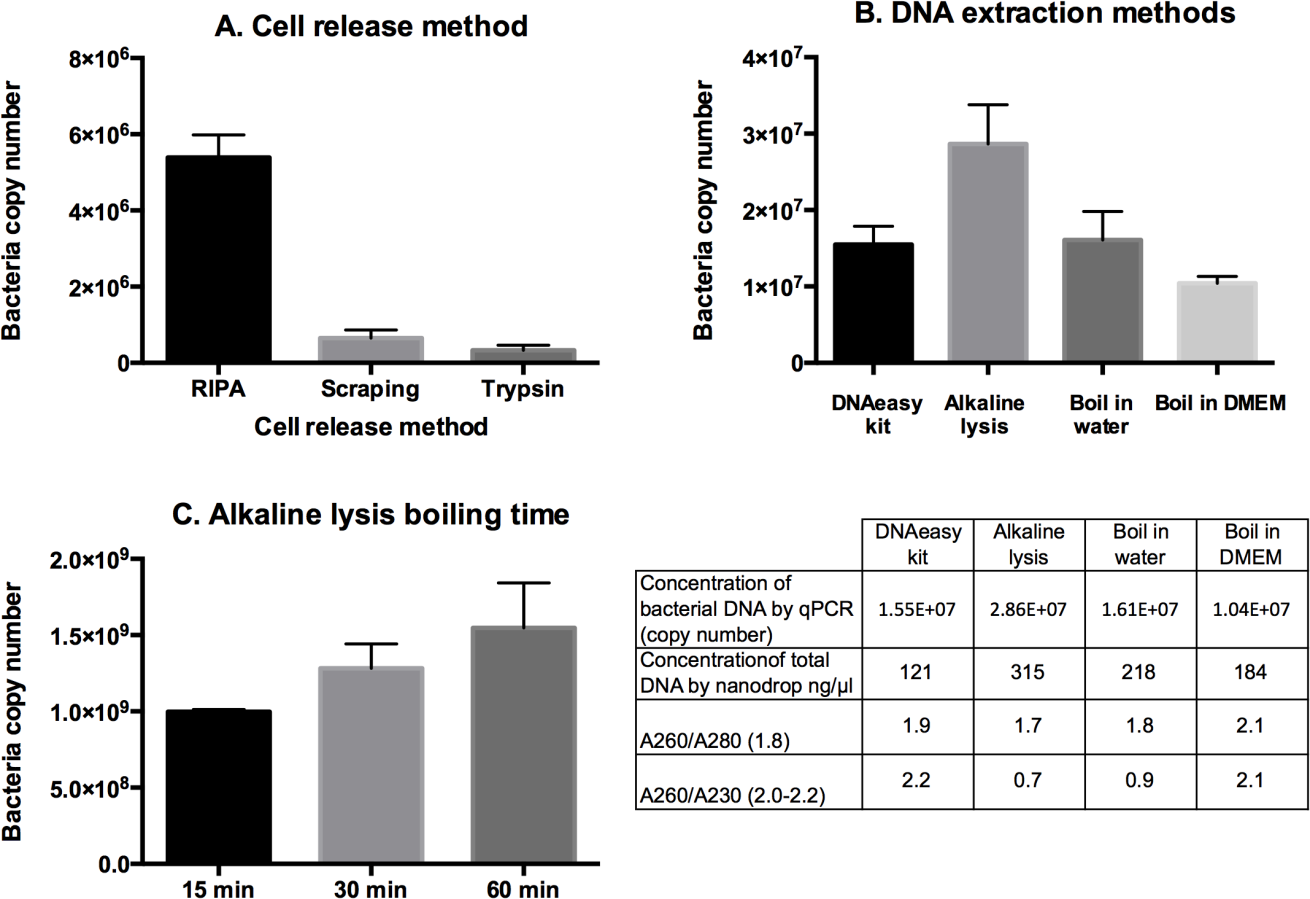
Optimising quantification of *O*. *tsutsugamushi* grown in cultured mammalian cells. Panel A. Levels of bacterial DNA as determined by qPCR, after releasing host cells from tissue culture flasks by different methods. Panel B. A comparison of DNA extraction methods. The graph shows the amount of *O*. *tsutsugamushi*-specific DNA detected by qPCR, and the table below shows the total concentration by qPCR or nanodrop analysis, the 260/280 absorption ratio and the 280/230 absorption ratio. Numbers in brackets indicate expected values for pure DNA. Panel C. Levels of bacterial DNA after boiling for different periods of time using the alkaline lysis DNA extraction method.

Growth assays were performed in 12-well or 24-well cell culture plates. We compared methods for removing and lysing the adherent L929 cells which had been infected with *O*. *tsutsugamushi*. Samples grown and infected under identical conditions were treated using RIPA cell lysis buffer, scraping using a sterile inoculating loop or release using trypsin-EDTA. All samples were then processed using the alkaline lysis DNA extraction method and it was found that treatment with RIPA buffer released approximately 3 times more bacteria than the other two methods (Fig 2A). We selected to use this method in all future experiments.

Different DNA extraction methods were compared (Fig 2B). Identical samples were extracted using a Qiagen DNAeasy kit, Alkaline lysis, boiling in water or boiling in cell growth media. Samples were adjusted to the same volume and quantified by qPCR. The alkaline lysis method resulted in the highest level of *O*. *tsutsugamushi* DNA, with the other three methods roughly equivalent. The DNAeasy kit, however, resulted in the highest purity DNA as assessed by nanodrop analysis (Fig 2, table). The appropriate method will therefore depend on individual requirements for cost, speed, purity and quantity. The alkaline lysis method is simple and cheap, and yields high levels of detectable bacterial DNA, and we selected to use it in all subsequent experiments described here.

Finally, we determined the effect of boiling time on the DNA yield produced by the alkaline lysis DNA extraction method (Fig 2C). We found that increasing boiling time released more bacterial DNA, but the differences were not very large. We used boiling times ranging between 15 min and 60 min in subsequent experiments, although this was kept constant within a single experiment.

### Optimisation of *O*. *tsutsugamushi* growth in cultured mammalian cells

**Fig 3:**
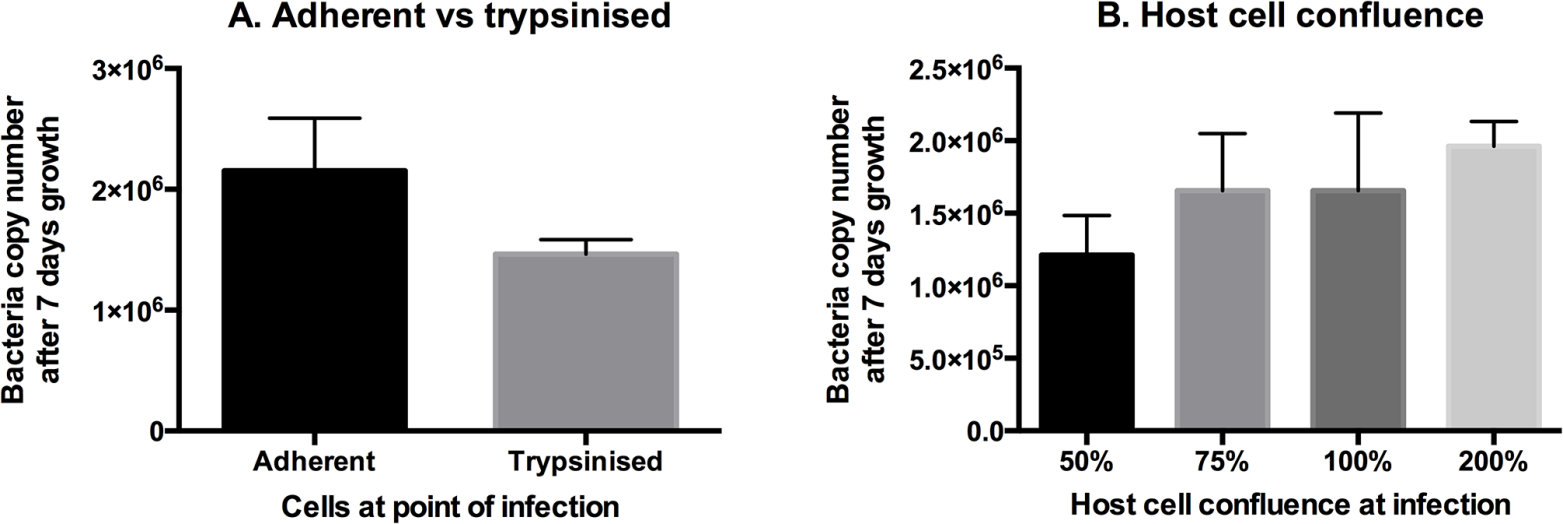
Optimising inoculation conditions for growth of *O*. *tsutsugamushi* in cultured L929 cells. Panel A. The growth of *O*. *tsutsugamushi* after infecting host cells in an adherent or trypsinised state. Panel B. The growth of *O*. *tsutsugamushi* after infecting host cells at different levels of confluence.

We compared the growth of *O*. *tsutsugamushi* after 7 days under a range of different growth conditions. First, we compared the effects of different inoculation conditions (Fig 3). It has previously been reported that *O*. *tsutsugamushi* can infect cells both in the adherent and trypsinised state [21]. We repeated this measurement and found a slight decrease in the efficiency of bacterial uptake when infecting newly trypsinised cells compared with adherent cells (Fig 3A). For routine work, therefore, we performed all infections on adherent cultured cells. However, this observation increases the potential for flexibility in future experimental designs as needed.

We measured the growth of bacteria in host cells that had been infected at different levels of confluence (Fig 3B). After 7 days the host cells had all grown to full confluence and any effect is likely to be dominated by a difference in efficiency of bacterial uptake upon infection. We found that host cell confluence had no strong effect on bacterial copy number after 7 days. The bacterial level in cells infected at 50% confluence was slightly lower than the others, but this can be accounted for by the fact that there were fewer host cells available for infection at time 0. From this we concluded that the host cell confluence had no strong effect on bacterial uptake and replication, and that this aspect of future workflow could be considered non-critical.

**Fig 4:**
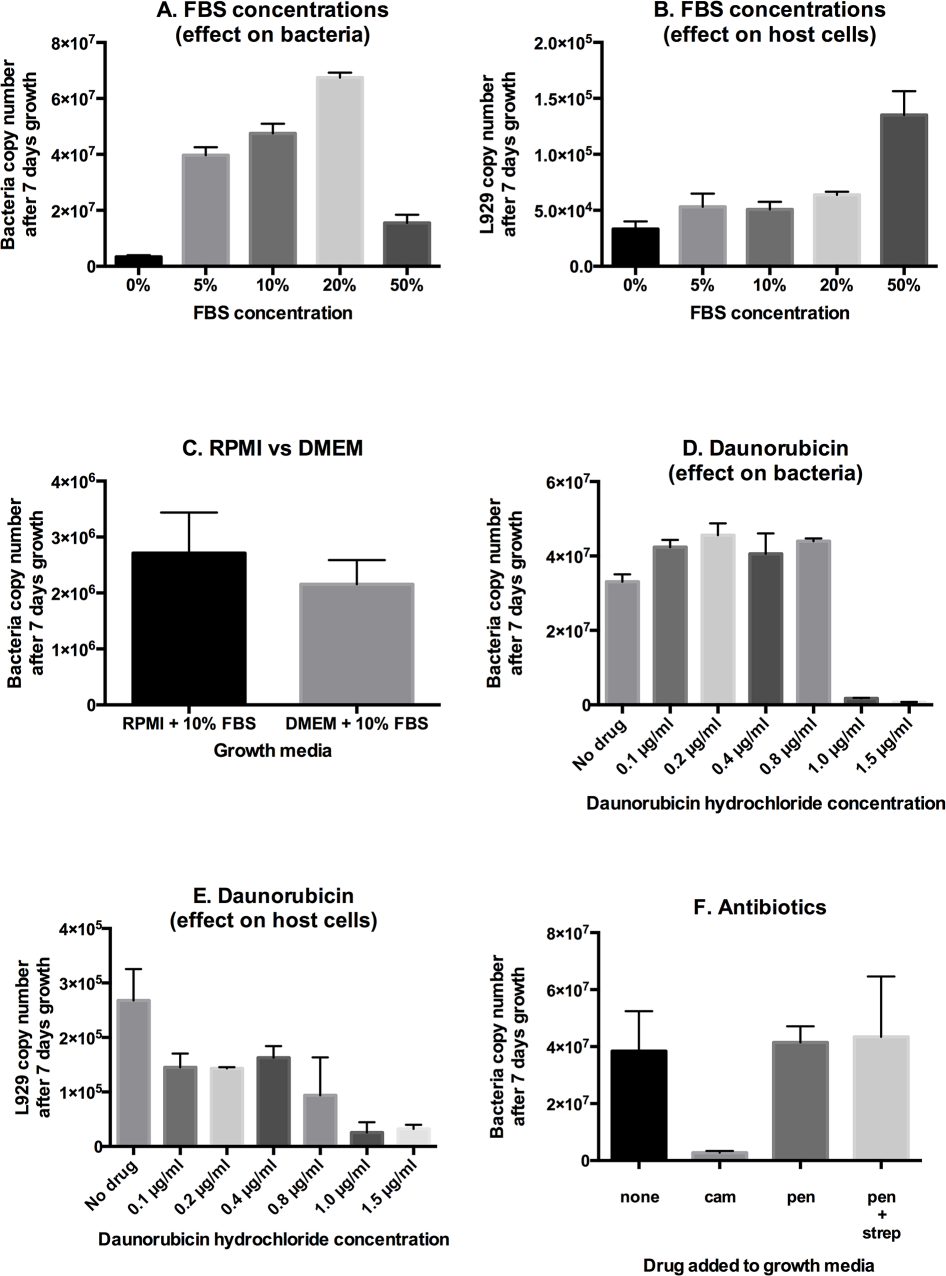
Optimising media conditions for growth of *O*. *tsutsugamushi* in cultured L929 cells. Panel A. The relationship between bacterial growth and FBS concentration. Panel B. The relationship between host cell (L929) growth and FBS concentration. Panel C. The growth of *O*. *tsutsugamushi* in RPMI or DMEM growth media. Panel D. The growth of *O*. *tsutsugamushi* in the presence of varying levels of daunorubicin. Panel E. The growth of L929 cells in the presence of varying levels of daunorubicin. Panel F. The growth of *O*. *tsutsugamushi* in the presence of different antibiotics. Antibiotic were used at the following concentrations: chloramphenicol (cam) 150 μg/ml, penicillin G (pen) 100 μg/ml (alone), penicillin G 125 μg/ml + streptomycin (strep) 200 μg/ml (combined).

Next, we compared the growth of *O*. *tsutsugamushi* grown in different growth media and with different supplements (Fig 4). We found that addition of fetal bovine serum (FBS) improved the growth of bacteria as shown previously [19]. We found that bacterial growth was increased when FBS was added at 5% and then further when added at 10% or 20%, but that addition of 50% FBS was inhibitory compared with lower concentrations. (Fig 4A). We also quantified the growth of L929 host cells in these experiments using a qPCR assay based on the mouse gene *cfd*, and found that growth in the presence of 50% FBS was increased around 3-fold compared with any of the lower FBS concentrations (Fig 4B). Therefore, the inhibition of FBS at high concentrations may be due to a reduction in bacterial replication in rapidly dividing host cells. Given the high cost of FBS we used 10% in all standard experiments. In cases where the inoculating level of bacteria is limiting, however, such as in clinical isolates, it may be advantageous to increase the level of FBS supplementation to 20%.

Mouse fibroblast (L929) cells can grow well in both RPMI and DMEM growth media, and we evaluated whether there would be any significant effect on bacterial copy number between the two (Fig 4C). We found no significant difference and therefore concluded that both would be suitable to support growth of *O*. *tsutsugamushi*. Daunorubicin hydrochloride is a drug that inhibits DNA and RNA synthesis in mammalian cells. This leads to a growth inhibition, and this has previously been shown to enhance the growth of bacteria in cultured mammalian cells [19,25]. A relationship between *O*. *tsutsugamushi* growth and host cell growth rate was also indicated in the FBS experiment, above. The optimum amount of daunorubicin to support bacterial growth varies between strains and host cell types, and we set out to determine the optimum concentration to support growth of *O*. *tsutsugamushi* in L929 cells (Fig 4D). Our results show a small increase in bacterial copy number after 7 days growth in the presence of 0.2, 0.4 and 0.8 μg/ml compared with no daunorubicin. This corresponds to a decrease in host cell replication (Fig 4E). At 1 μg/ml and 1.5 μg/ml bacterial growth was greatly reduced, and this is likely explained by excessive damage to host cells (Fig 4E). Whilst the small growth enhancement shown here may not be necessary for routine growth, this could be beneficial in cases where bacterial growth is difficult.

Antibiotics are sometimes added to cultured mammalian cells in order to avoid bacterial contamination. A combination of penicillin and streptomycin is frequently added to *O*. *tsutsugamushi* cultures for this purpose [26]. We measured the growth of *O*. *tsutsugamushi* in the presence of penicillin-streptomycin and found no effect on growth rate, supporting its use where required (Fig 4E). Chloramphenicol, which is active against *O*. *tsutsugamushi*, was used as a positive control and inhibited bacterial growth as expected [25,27].

### Optimising purification of live *O*. *tsutsugamushi* from mammalian host cells

For certain experiments it is desirable to purify bacteria from the host cells in which they are growing, for example to study growth in a different host cell type or to study the early stages of bacterial infection. This can be performed using percoll density gradient centrifugation [28], or by lysing host cells and separating released bacteria by centrifugation and filtration [29]. The latter approach was employed here. Since *O*. *tsutsugamushi* is an obligate intracellular bacterium this process will inevitably cause some damage to some fraction of the bacterial cells purified in this way. Our aim in this set of experiments was to quantify the loss of number and viability of bacteria purified under different conditions, in order to develop improved protocols for extracting bacteria from their host cells when required.

**Fig 5:**
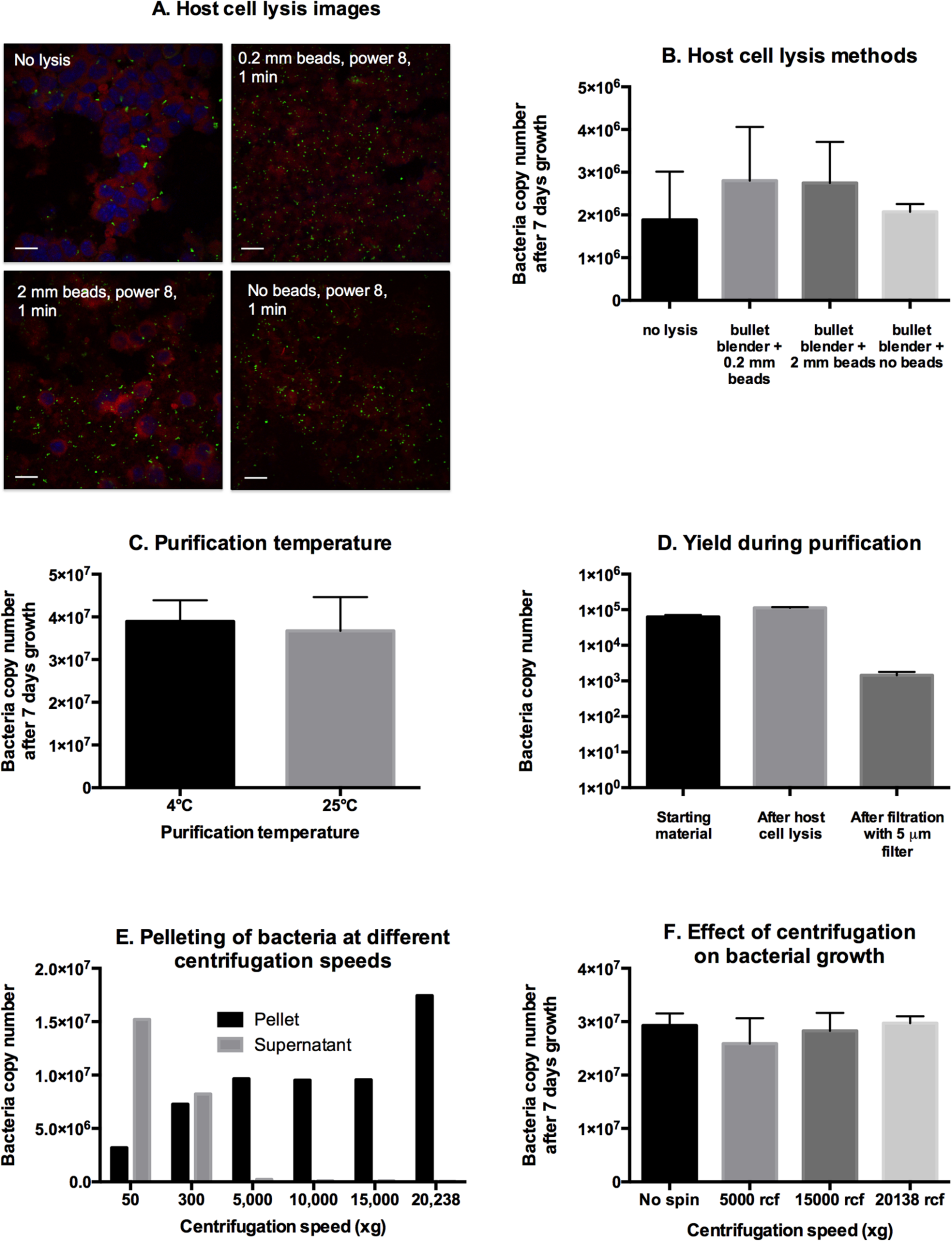
Optimising lysis of L929 host cells infected with *O*. *tsutsugamushi*. Panel A. Confocal fluorescence microscopy images showing the effect on host and bacterial cells of different lysis methods. Blue = nuclei (DAPI), red = host cells (Evans blue) and green = bacteria (Alexafluor 488-labelled antibody). Scale bar = 40 μm. Panel B. The effect of different host cell lysis methods on subsequent growth of bacteria. Panel C. The effect of purification temperature on subsequent growth of bacteria. Panel D. The yield of bacteria after different stages of purification. Panel E. the pelleting of bacteria after centrifugation at different g-forces. All samples were spun for 3 mins. Panel F. The effect of different centrifugation speeds on subsequent bacterial growth.

The first step in bacterial purification is host cell lysis. There are a number of ways in which this is achieved, with varying degrees of efficiency and bacterial damage. First, we used fluorescence light microscopy to study the physical effect of a range of different host cell lysis methods (Fig 5A and S1 Fig). We imaged both bacteria and host cells, and found that lysis using the bullet blender with either 0.2 mm glass beads, or no beads at all, led to efficient lysis of host cells and release of bacteria. Surprisingly, addition of 2 mm stainless steel beads appeared to inhibit host cell lysis under these conditions. Where the bullet blender instrument is not available, or where large volumes are required, vortexing for 1 min in the presence of 0.2 mm beads is the best option. Having identified efficient lysis methods, we wanted to measure the effect on bacterial viability of different methods of host cell lysis (Fig 5B). In order to do this we compared the growth of bacteria that had not been lysed, or lysed in a range of different methods. We found that the bacteria from lysed host cells were viable and demonstrated growth to a comparable level as bacteria from host cells that had not been lysed. It is important to note, however, that the uptake efficiency of bacteria from unlysed hosts is likely to be lower than that of purified bacteria, because they need to first exit the original, unlysed host cell before entering the new cell. Therefore we cannot truly compare the uptake and growth between the bacteria from cells that had been lysed, and those that had not. We can, however, conclude that bacterial purification by these methods has not resulted in massive loss of viability.

We compared the viability of bacteria that had been purified at 4°C and 25°C and found no significant difference (Fig 5C). We concluded, therefore, that future bacterial purifications could be conducted at room temperature with no effect on subsequent growth.

We quantified the yield of bacteria at different stages of purification (Fig 5D). After host cell lysis, samples were spun at 50 xg for 3 min to remove host cell debris. This resulted in no detectable loss of bacteria. Samples were then filtered using a 5 μm pore-size filter, and this resulted in a large loss of bacteria (about 90% loss). We concluded that this loss could be tolerated when a high degree of purification was desired (for example when moving between different host cell types) but that in other cases filtration could be excluded where high bacterial levels were a priority.

Finally, we measured the sedimentation of purified *O*. *tsutsugamushi* after centrifugation at different speeds (Fig 5E). A sample of purified bacteria was separated into 6 identical Eppendorf tubes, and these were each spun at different speeds. The supernatant was separated from the pellet and the levels of bacteria in each was quantified by qPCR. It was found that at low speeds (50 and 300 xg) there remained high levels of bacteria in the supernatant, but that at speeds of 5,000 xg and above, most of the bacteria were in the cell pellet. There was no difference in bacterial numbers in the pellets of samples spun at 5,000 xg, 10,000 xg and 15,000 xg, but the total amount almost doubled when sample speeds reached the maximum speed of 20,238 xg. From these observations we concluded that the majority of intact bacteria pelleted at 5,000 xg with no significant increase up to 15,000 xg but that at 20,238 xg small fractions of bacteria and free DNA were also pelleted. We then performed growth experiments in order to determine whether high centrifugation speeds had a negative effect on bacterial viability. We pelleted purified bacteria at different speeds and measured the growth after 7 days (Fig 5F). High pelleting speeds had no effect on subsequent growth of bacteria, and therefore we conclude that centrifugation speeds of 5,000–20,238 xg are appropriate for bacterial purification.

### Quantifying the effect of short-term storage on bacterial viability

In some cases it may be desirable to store isolated bacteria for short periods of time in order to perform certain experiments. It would therefore be advantageous to know exactly how much damage is caused to bacterial cells after storage under different conditions and for different periods of time.

**Fig 6:**
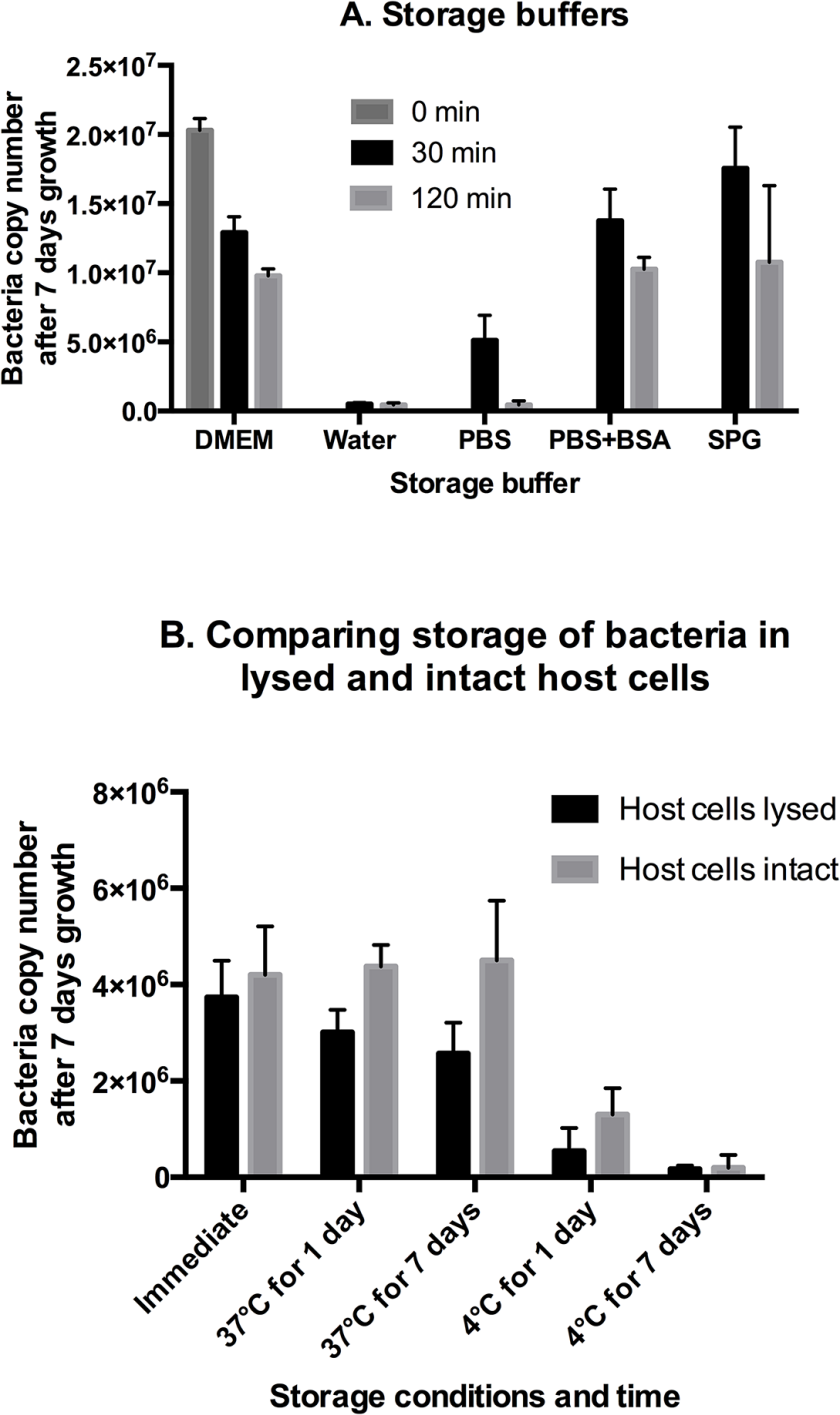
Quantifying the effect of short-term storage on bacterial viability. Panel A. Growth of *O*. *tsutsugamushi* after storage at room temperature for 30 min or 120 min in a range of different buffers. Panel B. Growth of *O*. *tsutsugamushi* after storage for 1 day or 7 days in lysed or intact host cells. In this experiment bacteria were stored in the growth media in which they were previously grown.

First, we measured the effect of storing purified bacteria in a range of different buffers for 30 mins or 120 mins at room temperature (Fig 6A). Bacteria were isolated from host cells (without filtering), resuspended in DMEM, water, PBS, PBS + 2% BSA, or SPG buffer, and stored at room temperature for 30 min or 120 min before being inoculated onto a lawn of L929s and grown for 7 days. As expected, it was found that water had the greatest negative effect on bacterial growth, probably due to the high osmotic stress to the cells. In support of this, the inhibitory effect of PBS could be somewhat relieved by the addition of BSA, which would increase the osmolarity of this solution. It was found that storage for 120 mins caused a much greater loss of bacteria than storage for 30 mins under all conditions. From this we concluded that *O*. *tsutsugamushi* is sensitive to solute osmolarity and that even a short incubation time caused a decrease in bacterial viability. This is not surprising because *O*. *tsutsugamushi* is an obligate intracellular bacterium that is unlikely to experience conditions of low osmolarity in its environment and is unlikely to spend any significant portion of its life cycle in a purified, cell-free state.

Second, we aimed to compare the stability of bacteria that had been purified from their host cells with bacteria that were stored within mammalian host cells (Fig 6B). The host cells were scraped from the cell culture flask and all samples were stored in spent culture media in sealed Eppendorf tubes. We compared the viability of purified and non-purified bacteria stored at 37°C or 4°C for 1 day or 7 days. We found that the bacterial viability went down slightly after storage for one day or 7 days at 37°C, and decreased more significantly upon storage at 4°C. In all cases the loss in purified cells was greater than that in intact cells, and in fact when bacteria were stored at 37°C in intact host cells no loss of viability was detected. This is likely to result from the presence of intact, live L929 cells stored at 37°C, even though samples were not grown in ideal culture conditions for the duration of the storage. From these data we concluded that storage in intact hosts was preferable to storage in lysed host cells and that long-term storage up to one week, where required, should be performed at 37°C.

### Optimisation of freezing conditions for *O*. *tsutsugamushi*

**Fig 7:**
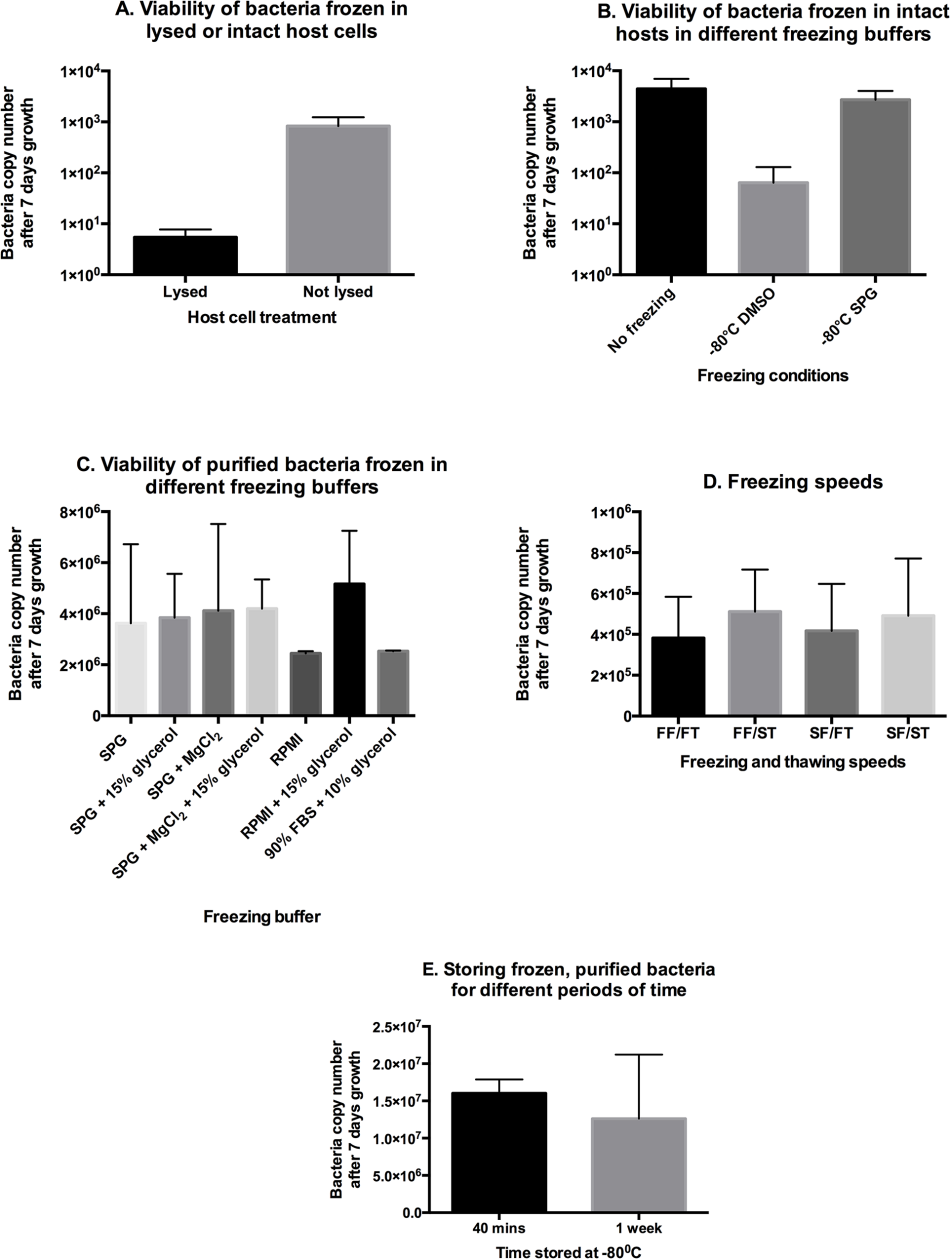
Optimising freezing conditions for preserving bacterial viability. Panel A. Comparing the growth of *O*. *tsutsugamushi* after being frozen in lysed or intact host cells, using SPG media. Panel B. Growth of bacteria after freezing in intact host cells in different buffers. Panel C. A comparison of different freezing buffers on the subsequent growth of purified bacteria and Panel D. Growth of *O*. *tsutsugamushi* after being frozen in SPG media at different freezing and thawing speeds. FF = fast freeze, SF = slow freeze, FT = fast thaw and ST = slow thaw. Panel E. Growth of *O*. *tsutsugamushi* after being frozen and stored for 40 mins or 1 week at-80°C. Bacteria were purified and stored in SPG buffer.

High quality cryopreservation of clinical isolates and laboratory-propagated bacteria is an important aim for all researchers engaged in scrub typhus research [30,31]. We aimed to quantify and optimise the effect of different freezing methodologies on bacterial viability. First, we compared the viability of bacteria cryopreserved in lysed or intact host cells. We observed a clear improvement when bacteria were frozen in intact host cells and recommend this practice where possible (Fig 7A).

We compared the viability of bacteria frozen in intact host cells using two different freezing buffers: FBS/DMSO or sucrose-phosphate-glutamate (SPG) buffer (Fig 7B). We found a clear benefit to using SPG freezing media, as has been recommended for other intracellular bacteria [29,32].

There are certain instances, however, where it is desirable to freeze purified bacteria. One example is when performing cell biology experiments where it would be advantageous to prepare a large batch of purified, validated bacteria to ensure consistency across future experiments. Therefore, we aimed to improve methods for freezing bacteria that had been purified from host cells.

First, we compared the viability of purified *O*. *tsutsugamushi* after freezing in a range of different buffers (Fig 7C). We found that SPG-based buffers exhibited a protective effect compared with RPMI alone, but that addition of 15% glycerol or MgCl_2_ to SPG had no additional benefit for bacterial viability. Freezing in the presence of RPMI + 15% glycerol, in contrast, gave the same degree of cryoprotection as freezing in SPG-based buffers.

Second, we measured the effect of freezing and thawing speed on bacterial viability (Fig 7D). We compared freezing in dry ice (fast) with freezing in isopropanol chambers (slow), and thawing in a 37°C water bath (fast) with thawing on ice (slow). Surprisingly, we found that the speed of freezing and thawing had no observable effect on bacterial viability. Therefore, we concluded that this process was not critical to the outcome of cryopreservation.

The above experiments were performed using short freezing times of 40 mins, as this enabled multiple experiments to be performed in one day and therefore different conditions to be directly compared. In order to determine whether this was sufficient time to measure the effects of freezing, we compared the growth of bacteria that had been stored frozen for 40 mins or 1 week. We found no significant different in recovery rates (Fig 7E). This study did not address the effect of extended freezing periods (months or years), nor the effects of storage in liquid nitrogen compared with a-80°C freezer.

### Conclusions

The aim of this study was to develop an evidence-based experimental toolkit to support future research into the important neglected human pathogen *O*. *tsutsugamushi*. Here, we have shown that a simplified growth assay can be used to compare the growth of this organism across a range of conditions, and this will be useful in other areas such as comparing the antibiotic sensitivity of a range of isolates.

Using an optimised, simplified growth assay we have shown that growth of *O*. *tsutsugamushi* in cultured mouse fibroblast (L929) cells can be improved by the addition of up to 20% FBS and 0.2–0.8 ug/ml daunorubicin, but that uptake and growth is not strongly affected by host cell media (RPMI/DMEM), the confluence of host cells at infection, or whether the host cells are adherent or recently trypsinised at the point of infection. These results suggest that flexibility in these parameters when growing *O*. *tsutsugamushi* in cultured cells can be tolerated.

It is frequently desirable to isolate live bacteria from the host cells in which they are growing, in order to perform experiments, for example on the cellular infection cycle. Here, we found that the efficiency of host cell lysis is sensitive to the time and power of lysis, and also to the bead type used. We show that effective host cell lysis has no significant effect on bacterial viability. We attempted to optimise the purification of *O*. *tsutsugamushi* from host cells, and found that the temperature of purification (4°C or room temperature) was not important, but that filtering using a 5 μm filter resulted in a loss of bacteria of about 1 order of magnitude. We found that pelleting speeds of 5,000 xg were sufficient to pellet the majority of purified *O*. *tsutsugamushi*.

We measured the effect of short-term storage on the viability of *O*. *tsutsugamushi*, since certain experiments may require periods of storage while other reagents are being prepared. We found that storage in a range of buffers resulted in a loss of viable bacteria over a short time frame (30–120 min) and that this could be reduced by the use of buffers of higher osmotic strength.

Finally, we compared the number of viable bacteria after cryopreservation across a number of different conditions. We found that preservation in intact host cells led to significantly larger numbers of viable bacteria than preservation of purified bacteria. However, where it is necessary to cryopreserve purified bacteria we found that different buffers could preserve bacteria to varying degrees. Throughout, we found that SPG buffer conferred the greatest protection against cryodamage but that the speeds of freezing and thawing had little effect on preserving bacterial viability.

Taken together, these data can be used to develop experimentally validated protocols for the purification, growth and storage of *O*. *tsutsugamushi*, and it is expected that some of these results will be transferable to other obligate intracellular bacteria.

## Supporting Information

S1 Fig Optimising lysis of L929 host cells infected with *O*. *tsutsugamushi*. Confocal fluorescence microscopy images showing the effect on host and bacterial cells of different lysis methods. Blue = nuclei (DAPI), red = host cells (Evans blue) and green = bacteria (Alexafluor 488-labelled antibody). Scale bar = 40 μm.
(TIFF)
