# Supplementary figures and images for: Improved Quantification, Propagation, Purification and Storage of the Obligate Intracellular Human Pathogen Orientia tsutsugamushi
Source: PLoS Negl Trop Dis. 2015 Aug 28;9(8):e0004009. doi: 10.1371/journal.pntd.0004009 (PMC4552649; doi:10.1371/journal.pntd.0004009)

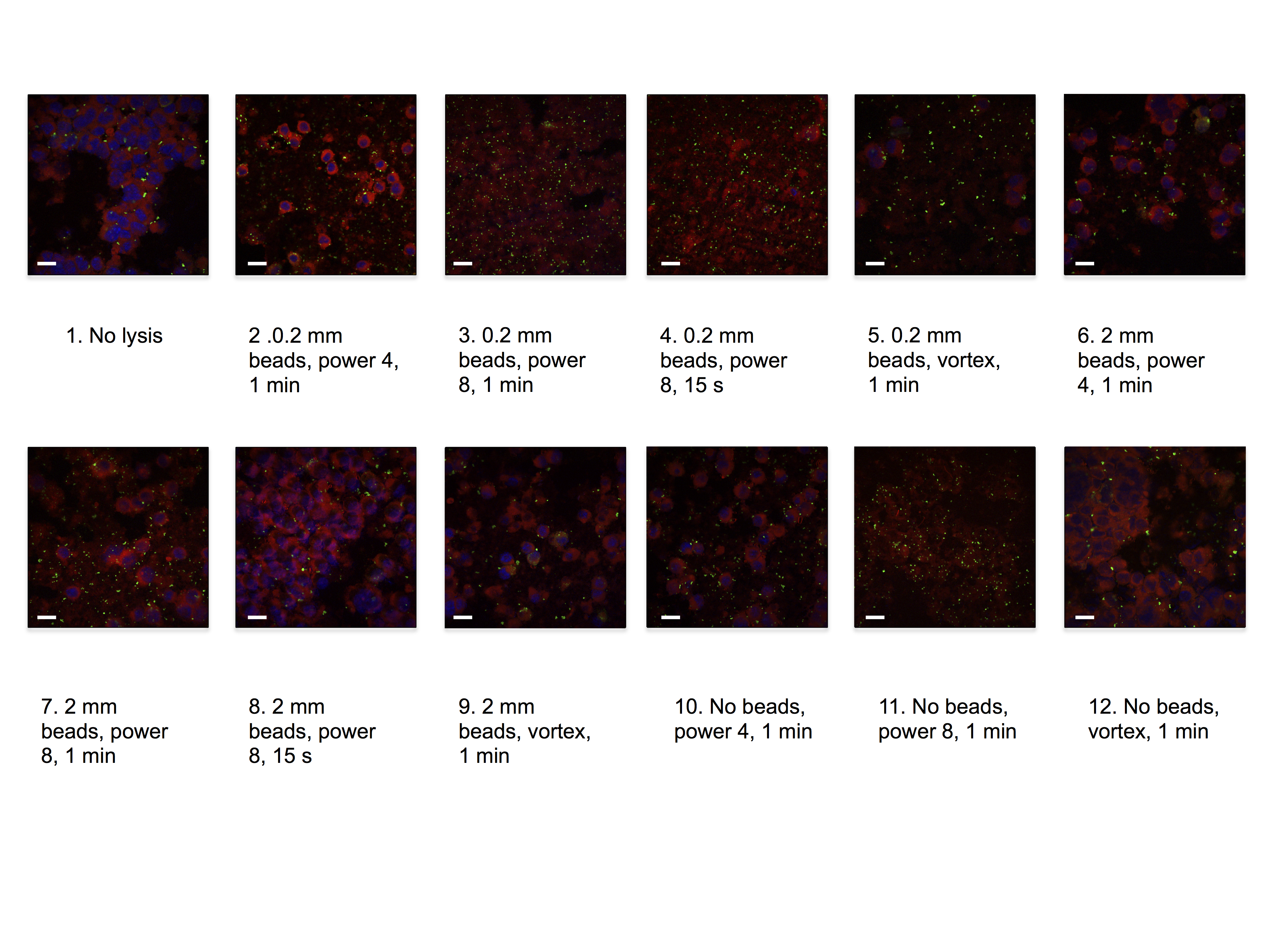

Supplement: S1 Fig — Confocal fluorescence microscopy images showing the effect on host and bacterial cells of different lysis methods. Blue = nuclei (DAPI), red = host cells (Evans blue) and green = bacteria (Alexafluor 488-labelled antibody). Scale bar = 40 μm. (TIFF) [file pntd.0004009.s001.tiff]
